# Atomically Dispersed Transition Metal-Nitrogen-Carbon Bifunctional Oxygen Electrocatalysts for Zinc-Air Batteries: Recent Advances and Future Perspectives

**DOI:** 10.1007/s40820-021-00768-3

**Published:** 2021-12-16

**Authors:** Fang Dong, Mingjie Wu, Zhangsen Chen, Xianhu Liu, Gaixia Zhang, Jinli Qiao, Shuhui Sun

**Affiliations:** 1 Institut National de La Recherche Scientifique (INRS)-Centre Énergie Matériaux Télécommunications, Varennes, QC J3X 1P7 Canada; 2 Key Laboratory of Materials Processing and Mold, Ministry of Education, Zhengzhou University, Zhengzhou, 450002 People’s Republic of China; 3 State Key Laboratory for Modification of Chemical Fibers and Polymer Materials, College of Environmental Science and Engineering, Shanghai Innovation Institute for Materials, Donghua University, Shanghai, 201620 People’s Republic of China; 4 Engineering Research Center of Nano, Geomaterials of Ministry of Education, China University of Geosciences, Wuhan, 430074 People’s Republic of China

**Keywords:** Atomically dispersed metal-nitrogen-carbon, Oxygen evolution reaction (OER), Oxygen reduction reaction (ORR), Bifunctional oxygen electrocatalysts, Zinc-air batteries (ZABs)

## Abstract

Rechargeable zinc-air batteries (ZABs) are currently receiving extensive attention because of their extremely high theoretical specific energy density, low manufacturing costs, and environmental friendliness. Exploring bifunctional catalysts with high activity and stability to overcome sluggish kinetics of oxygen reduction reaction and oxygen evolution reaction is critical for the development of rechargeable ZABs. Atomically dispersed metal-nitrogen-carbon (M-N-C) catalysts possessing prominent advantages of high metal atom utilization and electrocatalytic activity are promising candidates to promote oxygen electrocatalysis. In this work, general principles for designing atomically dispersed M-N-C are reviewed. Then, strategies aiming at enhancing the bifunctional catalytic activity and stability are presented. Finally, the challenges and perspectives of M-N-C bifunctional oxygen catalysts for ZABs are outlined. It is expected that this review will provide insights into the targeted optimization of atomically dispersed M-N-C catalysts in rechargeable ZABs.

## Highlights

- General principles for designing atomically dispersed metal-nitrogen-carbon (M–N-C) are briefly reviewed.
- Strategies to enhance the bifunctional catalytic performance of atomically dispersed M–N-C are summarized.
- Challenges and perspectives of M–N-C bifunctional oxygen catalysts for Rechargeable zinc-air batteries are discussed.

## Graphical abstract

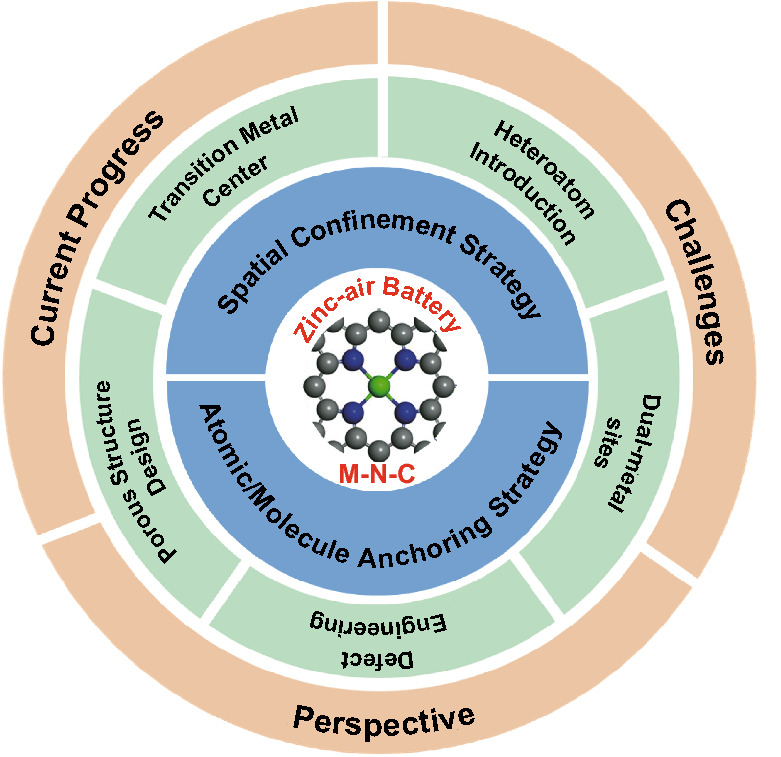

## Introduction

To alleviate the dependence on traditional fossil fuels and reduce environmental degradation, significant efforts have been focused on the development of harvesting, conversion, and storage of low-cost and environmentally friendly renewable energy [1–4]. As one of the most promising next-generation energy storage devices, rechargeable zinc-air batteries (ZABs) are considered to be potentially used in electric vehicles, flexible wearable electronic devices, etc., due to their high theoretical energy density (1086 Wh kg^−1^), high cell voltage (1.66 V), good safety, low cost, and environmental friendliness [5–9]. While there have been considerable advances in recent years, ZABs still face some critical challenges. The absence of a reliable and effective bifunctional air electrode has been the most significant impediment to its practical application [10–14]. The performance of the ZABs is highly dependent on the catalytic performance of bifunctional oxygen electrocatalyst on the air electrode, in which kinetically sluggish oxygen reduction reaction (ORR, discharge process) and oxygen evolution reaction (OER, charge process) proceed during discharge–charge processes [15–17]. Therefore, bifunctional electrocatalysts efficient in both ORR and OER are the keys to high-performance rechargeable ZABs. The potential gap (ΔΕ = E_j10_—E_1/2_) between E_j10_ (the potential to reach an OER current density of 10 mA cm^−2^) and E_1/2_ (the potential to reach half of the ORR limiting current density) is a crucial parameter for evaluating bifunctional catalytic performance. Smaller ∆E value amounts to a lower efficiency loss and preferable catalytic performance during reversible oxygen catalysis [10, 18].

ORR and OER generally require different catalysts because of the complicated mechanisms. Noble-metal catalysts with high catalytic activity for ORR (e.g., Pt/C) and OER (e.g., IrO_2_, RuO_2_) can greatly accelerate their sluggish kinetics [19]. However, they cannot meet the requirements of bifunctional catalytic activity in rechargeable ZABs because they are merely efficient in one of the two reactions (ORR or OER) [20–25]. It is common to mix the ORR-active and OER-active components to obtain a bifunctional catalyst. Unfortunately, the simple mixing of noble-metal catalysts is not always sufficient to achieve precise catalytic selectivity for ORR and OER. The insufficient bifunctional catalytic activity, inferior durability, and high cost severely hinder the practical application of rechargeable ZABs [26–30]. As earth-abundant and low-cost alternatives to noble metal-based electrocatalysts, transition metal (TM)-based materials and carbon-based materials have recently attracted extensive research attention [17, 31–38]. Developing effective strategies (e.g., surface functionalization, structure engineering, heteroatoms doping, and creating defect) to improve their intrinsic activity and increase exposed effective active sites play a vital role in boosting their bifunctional activity and stability [31, 39–44]. TM oxides or hydroxides are reported to be one of the most effective OER electrocatalysts, exhibiting a higher current density and better stability compared with metal-free carbon materials [45, 46]. Nevertheless, they have poor conductivity and scarcely catalyze ORR efficiently. Meanwhile, as efficient metal-free alternatives to noble-metal catalysts for ORR, nanostructured carbon-based materials with high electrical conductivity and high surface area (e.g., carbon nanotubes, porous carbon, and N-doped graphene), have also drawn wide research attention [26, 31, 45, 46]. In comparison, the majority of these metal-free catalysts exhibit insufficient OER activity [45]. Intriguingly, carbon materials can serve as conductive skeletons and active components of bifunctional catalysts through effective modifications, such as heteroatoms doping into carbon lattice, manufacturing defects, and hybridization with other active species [47]. When carbon materials are doped with TM atoms, they possess both high electrical conductivity of carbon materials and high intrinsic activity of metal-based active sites [48–50]. Furthermore, the TMs in such catalysts can also significantly improve the graphitization of carbon materials, providing them with excellent protection from corrosion and accumulation during electrochemical reactions [23, 51]. Therefore, incorporating the TMs into the carbon materials to obtain highly efficient bifunctional catalysts for ORR and OER has aroused extensive interest and exploration in rechargeable ZABs.

Transition metals (e.g., Co, Fe, Ni, Mn) and nitrogen (N) co-doped carbons (M-N-C), as promising candidates for ORR catalysts, have been widely studied [48]. Although the OER activity is relatively inadequate, their adjustable coordination structures and electronic environments are advantageous for enhancing the OER performance. For instance, the effective regulation of electronic configurations of metal centers and the introduction of active sites are effective to boost their catalytic activity. Therefore, the M-N-C catalysts are promising to be used as highly efficient bifunctional oxygen catalysts [1, 21, 22, 31, 52–55]. However, inhomogeneous microstructures and the uncontrollable agglomeration of active components usually result in inadequate exposure of active sites and poor electron/mass transport. Consequently, the gradual inactivation of ORR and OER during the long-term charge/discharge leads to the loss of battery performance [51, 56, 57]. Atomically dispersed M-N-C electrocatalysts bring out new opportunities to oxygen electrocatalysis. Their ultrahigh intrinsic reactivity, high atomic utilization, favorable electronic conductivity, and ion transport endowed by the carbon skeleton can further enhance electrocatalytic performance [58–60].

**Fig. 1:**
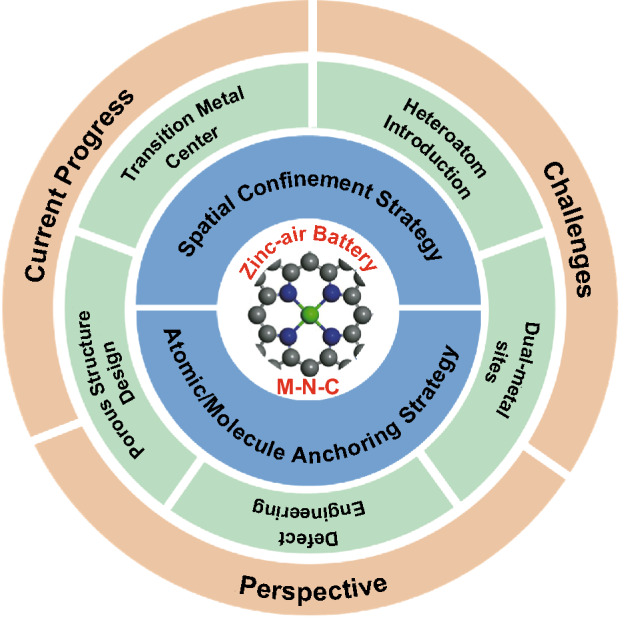
Schematic diagram of general design principles and strategies aimed at enhancing the bifunctional catalytic activity of atomically dispersed M-N-C electrocatalysts in this review

In this review, we overview atomically dispersed M-N-C bifunctional oxygen electrocatalysts for rechargeable ZABs (Fig. 1, Table 1). Herein, we first introduce general design principles for atomically dispersed M-N-C, including atomic/molecule anchoring and spatial confinement strategies. Then we particularly focus on how to enhance their bifunctional catalytic performance mainly including: i) identifying the catalytic performance of different TM centers; ii) regulating the coordination environment of atomically dispersed M-N_x_ sites by introducing heteroatoms, defects, and bimetals, and iii) designing favorable structures with hierarchical pores structure. Finally, challenges and prospects of developing atomically dispersed M-N-C electrocatalysts with bifunctional ORR/OER activity are outlined to provide a comprehensive understanding.

**Table1:** Comparison of bifunctionality and Zn-air battery performances of different kinds of atomically dispersed M-N-C electrocatalysts

Catalysts	Types	E_1/2_ vs. RHE (V)	E_j=10_ vs. RHE (V)	ΔΕ (V)	Peak power density (mW cm^−2^)	Specific capacity (mAh g_Zn_^−1^)	Cycling stability	Refs.
Fe-NC/rOCNT	Fe-N-C	0.865 (0.1 M KOH)	1.672 (0.1 M KOH)	0.807	182	–	1000 h (10 mA cm^−2^)	[48]
H-ZIF-Fe-SCN	Fe-N-C	0.889 (0.1 M KOH)	1.69 (0.1 M KOH)	0.78	158.8	–	80 cycles (10 mA cm^−2^)	[61]
FeN_x_-PNC	Fe-N-C	0.86 (0.1 M KOH)	1.635 (0.1 M KOH)	0.775	118	–	220 cycles (40 h)(5 mA cm^−2^)	[11]
SCoNC	Co-N-C	0.91 (0.1 M KOH)	1.54 (0.1 M KOH)	0.63	194	690	20 h (5 mA cm^−2^)	[62]
Co/NHPC_150/800_	Co-N-C	0.85 (0.1 M KOH)	1.5 (0.1 M KOH)	0.65	425	–	1920 cycles (320 h) (5 mA cm^−2^)	[8]
CoNC@LDH	Co-N-C	0.84 (0.1 M KOH)	1.74 (0.1 M KOH)	0.63	173	800	3630 cycles (10 mA cm^−2^)	[10]
Fe-NSDC	Heteroatom coordination	0.84 (0.1 M KOH)	1.64 (0.1 M KOH)	0.80	225.1	740.8	400 cycles (4 mA cm^−2^)	[63]
Co–N,B-CSs	Heteroatom coordination	0.83 (0.1 M KOH)	1.66 (0.1 M KOH)	0.83	∼100.4	–	128 cycles (12 h) (10 mA cm^−2^)	[64]
S,N-Fe/N/C-CNT	Heteroatom coordination	0.85 (0.1 M KOH)	1.60 (0.1 M KOH)	0.75	102.7 m	–	100 cycles (5 mA cm^−2^)	[65]
FeN_4_CB	Heteroatom coordination	0.84 (0.1 M KOH)	1.580 (1 M KOH)	0.74	177	800	220 h (10 mA cm^−2^)	[66]
Co, N, P-PCNS	Heteroatom coordination	0.87 (0.1 M KOH)	1.549 (1 M KOH)	0.679	101.3	–	2,000 cycles (40 h) (10 mA cm^−2^)	[67]
Fe/N-G-SAC	Defect engineering	0.89 (0.1 M KOH)	1.60 (0.1 M KOH)	0.71	120	–	240 cycles (10 mA cm^−2^)	[23]
FePc@N,P-DC	Defect engineering	0.903 (0.1 M KOH)	1.56 (0.1 M KOH)	0.66	~ 120	–	200 cycles (~ 50 h) (5 mA cm^−2^)	[68]
Fe/Ni(1:3)-NG	Dual-metal sites	0.842 (0.1 M KOH)	1.709 (0.1 M KOH)	0.857	164.1	824.3	120 h (5 mA cm^−2^)	[49]
Co1-PNC/Ni1-PNC	Dual-metal sites	0.88 (0.1 M KOH)	1.62 (1 M KOH)	0.74	252	874	45 h (10 mA cm^−2^)	[69]
CoNi-SAs/NC	Dual-metal sites	0.76 (0.1 M KOH)	1.57 (0.1 M KOH)	0.81	101.4	750.9	95 cycles (5 mA cm^−2^)	[22]
Fe–NiNC-50	Dual-metal sites	0.84 (0.1 M KOH)	1.57 (1 M KOH)	0.73	~ 220	752.14	100 h (2 mA cm^−2^)	[21]
Ni_66_Fe_34_-NC	Dual-metal sites	0.85 (0.1 M KOH)	1.699 (0.1 M KOH)	0.849	140.1	765.5	334 h (20 mA cm^−2^)	[53]
NiN_4_/GHSs/Fe-N_4_	Dual-metal sites	0.83 (0.1 M KOH)	1.62 (0.1 M KOH)	0.79	777.6	–	600 cycles (~ 200 h) (10 mA cm^−2^)	[29]
meso/micro-FeCo-N_x_-CN	Structure design	0.886 (0.1 M KOH)	1.666 (0.1 M KOH)	0.78	150	–	~ 20 h (20 mA cm^−2^)	[70]
Co–N_x_/C NRA	Structure design	0.877 (0.1 M KOH)	1.53 (6 M KOH)	0.65	193.2	–	80 h (50 mA cm^−2^)	[71]
Fe_0.5_Ni_0.5_@N-GR	Structure design	0.83 (0.1 M KOH)	1.38 (1 M KOH)	0.55	85	940	18 h (10 mA cm^−2^)	[72]
NeCNC-900	Structure design	0.90 (0.1 M KOH)	1.52 (0.1 M KOH)	0.645	68.2	–	1200 cycles (200 h) (10 mA cm^−2^)	[73]

## General Principles for Designing Atomically Dispersed M-N-C Catalysts

The interaction between metal atoms and the carbon matrix is weak, which makes individual atoms stabilize on the carbon matrix quite difficult. The metal center usually needs to cooperate with other atoms on the carrier to maintain stability. N-doped porous carbon as a carrier has a high specific surface area, abundant pores, and high N content, which is considered one of the most widely used substrates for stabilizing single metal atoms [74–76]. On the one hand, N is more electronegative and reactive than carbon, which makes the interactions between the metal and N atoms stronger [75]. The introduction of N into the carbon lattice by chemically bonding with adjacent carbon atoms plays a crucial role in anchoring isolated metal atoms [5, 31]. On the other hand, N atoms in the carbon network cannot only adjust the electronic structure of adjacent carbon atoms to make charge redistribution but also reduce the adsorption energy of oxygen-containing substances. It was identified that pyridinic N, graphitic N, and regulated carbon atoms are important sources of catalytic activity [5, 16, 31]. In general, the metal atom forms M-N_4_ by coordinating with four N atoms such as pyridine and pyrrole N, which can influence the adsorption efficiency of the reactant on the surface of the catalyst and ultimately affect the catalytic activity, selectivity, and stability toward ORR and OER [59, 77].

General approaches for preparing atomically dispersed M-N-C catalysts include top-down and bottom-up techniques [78]. (1) Top-down synthesis technology: nanomaterials or organic polymer precursors containing TM, N, and C are uniformly organized and transformed into the desired structures with highly effective catalytic active sites, such as pyrolysis [79–81]. Pyrolysis of the corresponding precursors containing carbon, nitrogen, and TMs is the traditional method for preparing M-N-C materials [58, 82]. High-temperature calcination can transform TM particles or blocks into single atoms when combined with N-doped porous carbon [75]. However, the generated single metal atoms tend to gather in clusters, preventing them from being dispersed on the carrier at the atomic level, which makes the single-atom active species co-exist with a large number of metallic particles [78, 82]. Inhibiting the agglomeration of metal atoms and directly converting the metal atoms into the active part of M-N are the keys to preparing atomically dispersed M-N-C catalysts [75]. The method has the advantages of easy operation, inexpensive use of raw materials and equipment. However, it is a huge challenge to avoid the formation of nanoparticles and nanoclusters while increasing the loading of single-atom metals. (2) Bottom-up synthesis technology: the metal precursors are adsorbed, reduced, and confined on supports, such as atomic layer deposition (ALD), wet chemical synthesis, and photochemical reduction. Due to excellent controllability, ALD is a widely used strategy for manufacturing carbon-based single-atom metal catalysts by continuous deposition of monatomic layers. However, the high cost of equipment and low catalyst yield limit their large-scale industrial applications. The wet chemical method, as a simple and easy preparation method without special equipment, is more widely used in the synthesis of carbon-based single-atom metal catalysts. It is vital to accurately control the reaction kinetics of wet chemical synthesis to achieve the formation of single metal atoms and to avoid the aggregation and dissolution of single metal atoms [83–85]. Specific methods of synthesis in detail have been continuously summarized by several groups [60, 62, 78, 82, 86, 87].

**Fig. 2:**
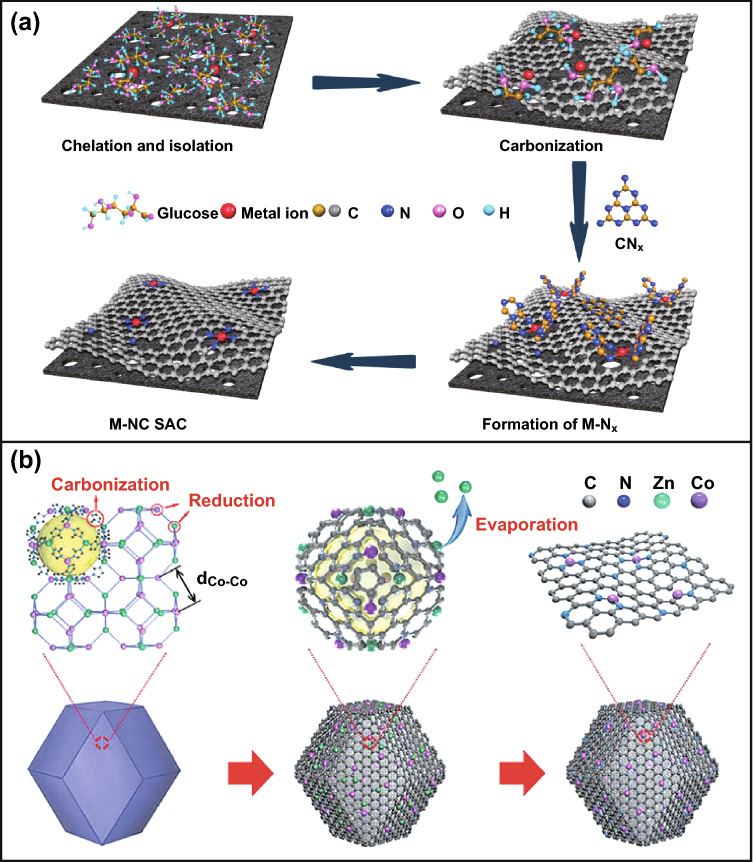
**a** The atomic/molecule anchoring strategy for the synthesis of M-N-C [88]. Copyright 2019, Nature Publishing Group. **b** Spatial confinement strategy for the formation of Co SAs/N-C [89]. Copyright 2016, Wiley–VCH

Increasing atomically dispersed metal loading is necessary to improve the intrinsic activity of M-N-C materials [78]. General principles for designing atomically dispersed M-N-C catalysts can be summarized into atomic/molecule anchoring strategy and spatial confinement strategy [82]. The former uses the unsaturated coordination and defects existing in the N-doped carbon carrier to act as “traps” to anchor and capture TM atoms/molecule through an enhanced charge transfer mechanism. Moreover, the chelation reaction between N-containing molecules and metal ions provides another effective way to establish strong M-N bonds [78, 82]. In general, anchoring metals by atom or molecule forms (Fig. 2a) is considered one of the most promising and successful ways for synthesizing atomically dispersed metal catalysts due to the ease of operation and the possible mass production. In addition, the defect capturing strategy also makes it possible to adjust the loading of atomically dispersed metals by controlling the concentration of defects. Recently, space-constrained strategy (Fig. 2b), as an effective way to subtly control spontaneous aggregation of atomic metal, has received more attention than the atomic/molecule anchoring strategy [89]. The stability and activity of the catalyst can be enhanced by a coordination structure that fixes a single metal atom in situ at its stable point. Metal–organic frameworks (MOFs) are the space-constrained precursors with M-N coordination that can be converted into M-N-C active sites in situ. They are stably coordinated with metal ions through chemical bonds, which adequately prevent the migration of metal atoms [78]. Encouraged by this idea, combined with their large specific surface area, adjustable pore size, rich-redox active metal centers, and diverse functions, MOFs have been considered as some of the most promising precursors to serve as self-sacrificial templates for the synthesis of atomically dispersed M–N-C bifunctional catalysts [16, 43, 90–92].

## Strategies to Enhance Bifunctional Activity of Atomically Dispersed M-N-C Catalysts

### Transition Metal Center

The current research on M-N-C catalysts is mainly focused on Fe, Ni, Co, or Mn-doped carbon material catalysts [93–98]. In particular, Fe-N-C materials receive the most attention due to their highest theoretical ORR activity. Central TM atoms are generally considered to play the most important roles in determining catalytic activity in M-N-C. Therefore, selecting the appropriate central metal atom is the most direct and effective strategy for regulating the intrinsic catalytic activity [97].

**Fig. 3:**
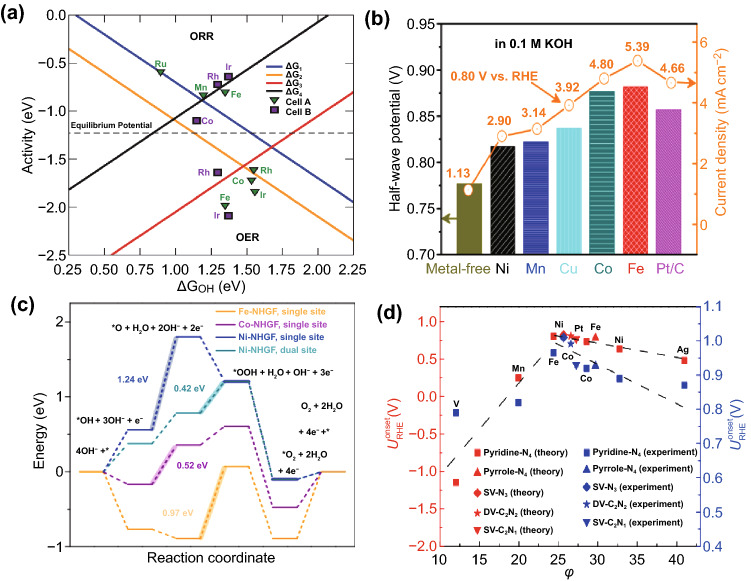
**a** Volcano plot for the ORR (up) and OER (below) [99]. Copyright 2011, Royal Society of Chemistry. **b** Half-wave potential and current density of different catalysts in 0.1 M [100]. Copyright 2014, American Chemical Society. **c** Free energy diagram at 1.23 V for OER over different catalysts [101]. Copyright 2018, Nature Publishing Group. **d** Comparison of experimental and theoretical onset potentials for ORR [96]. Copyright 2018, Nature Publishing Group

Based on density functional theory (DFT), Rossmeisl et al. found that the active sites composed of four N atoms and TM atoms in graphite materials were not only active for ORR but also active for OER [99]. From the volcano plot for the ORR (up) and OER (below) in Fig. 3a, it shows that Fe, Ir, Mn, can effectively catalyze ORR, while OER can be catalyzed by the active sites of Co. Considering that most M-N-C electrocatalysts can only provide a single function of ORR or OER, the design of bifunctional atomically dispersed M-N-C electrocatalysts for ORR and OER has a high value for the development of rechargeable ZABs [58]. Peng et al. studied the effect of different metal atoms in the center of the doped carbon catalysts on their ORR activity. It demonstrates that the ORR activity is ranked as Fe > Co > Cu > Mn > Ni in Fig. 3b, which is consistent with the order of their active N contents. The superior performance enhancement of the TMs was considered to be the combined effect of the content of nitride/active nitride, metal residue, and surface area and pores, rather than any one of them alone [100]. Zheng et al. also discovered that the intrinsic ORR activity follows the order of Fe > Co > Mn > Ni [98]. According to simulation and experimental verification based on theoretical simulation, Fei et al. confirmed that the OER activities of MN_4_C_4_ moieties follow the trend Ni > Co > Fe. The calculated energy diagrams of the OER that indicates the rate-determining step (RDS) with the values of the limiting energy barrier labeled are presented in Fig. 3c [101].

The trial-and-error method of determining which center metal atoms have the highest intrinsic electrocatalytic activity is common but still challenging, primarily due to the lack of a general design principle to guide the scanning [96, 97]. Xu et al. proposed a general design principle based on the descriptor φ, which directly related the catalytic activity to the electronegativity and coordination number of active metal centers and the nearest neighbor atoms [96]. It can serve as a general descriptor for predicting the catalytic performance of ORR/OER/HER to reveal the structure of the active center and the surrounding electronic environment. The experimental and theoretical ORR onset of atomically dispersed TM atoms supported on graphene versus the descriptor φ is shown in Fig. 3d. The results indicate that the experimental results match the theoretically predicted relative order of catalytic activity. Furthermore, the optimal active center can also be predicted using this descriptor, such as Fe-pyridine/pyrrole-N_4_, Co-pyrrole-N_4_, and Mn-pyrrole-N_4_ for efficient ORR, OER, and HER, respectively.

**Fig. 4:**
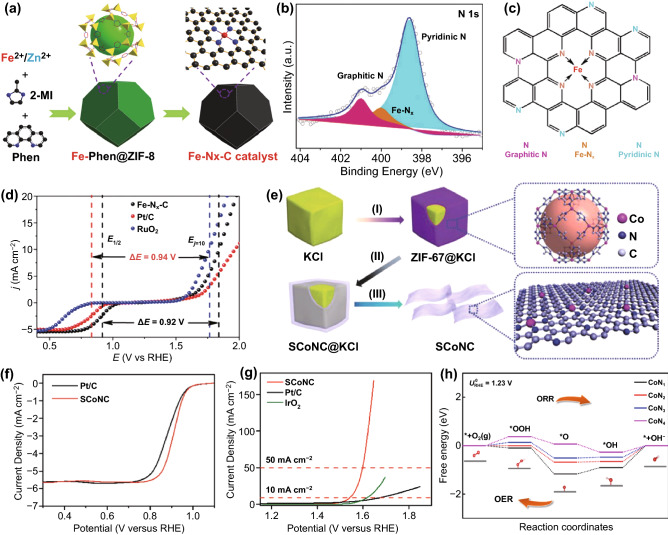
**a** The synthetic procedure of Fe-N_x_-C. **b** XPS spectrum of N 1 s. **c** The N species in the carbon framework. **d** Overall polarization curves of Fe-N_x_-C, commercial Pt/C, and RuO_2_ in 0.1 M KOH [102]. Copyright 2019, Wiley-VC. **e** The synthetic procedure of the SCoNC catalysts. **f** ORR and **g** OER polarization curves for the SCoNC and commercial catalysts in 0.1 M KOH. **h** The free energy diagram for ORR and OER of Co-N_x_ (x = 1, 2, 3, 4) [62]. Copyright 2019, Wiley–VCH

The best choice of the central metal atom for the bifunctional M-N-C catalyst is still controversial, even with the guidance of the identification of experiments and design principles. Considering that OER and ORR have different requirements for active sites, the biggest challenge is to concentrate the active sites for catalyzing both ORR and OER on the same M-N-C catalyst. Besides, different TM centers also lead to different structures, specific surface areas, and different active N content and distribution of N-doped carbon materials [31, 100]. Therefore, selecting a suitable metal center and adjusting their electronic configuration are the keys to optimizing good bifunctional performance [103]. Han et al. prepared a well atomically dispersed Fe-N_x_-C catalyst by encapsulating Fe-Phen species in the nanocages during the growth of ZIF-8 (Fig. 4a) [102]. From the XPS spectrum (Fig. 4b), pyridinic N (398.6 eV), Fe-N_x_ (400.0 eV), and graphitic N (401.0 eV) exist in the Fe-N_x_-C catalyst. From the schematic representation of the N-binding states in Fig. 4c, the pyridine N exists in the form of Fe-N_x_ as coordination for iron atoms. In addition, the graphite N has a positive effect on the geometric structure and electronic structure of the carbon skeleton, increasing the limiting current density of ORR. As a bifunctional catalyst, the ΔΕ of Fe-N_x_-C catalyst is around 0.92 V, which is better than that calculated by separate noble metal catalysts of Pt/C and RuO_2_ (ΔΕ = 0.94 V). As shown in Fig. 4d, the smaller ΔΕ of Fe-N_x_-C catalyst is mainly attributed to the excellent ORR activity; however, the OER activity of the Fe-N_x_-C catalyst is insufficient, compared with RuO_2_. The atomically dispersed Fe-N_x_-C electrocatalyst may function as a promising bifunctional catalyst by further improving the OER performance.

The extremely limited concentration of metal atoms (usually less than 1.5%) must be associated with N-based carbon supports to prevent agglomeration and ensure atom dispersion, which frequently hinders promoting catalytic efficiency [64]. The graphene-like carbon support with monodispersed cobalt atoms has been reported by Wu et al. [62]. The catalyst (SCoNC) was obtained by pyrolysis of KCl particles wrapped with a Co-based organic framework (ZIF-67), which achieved a dramatically high Co atoms concentration (≈15.3%) (Fig. 4e). In contrast to the benchmark Pt/C catalyst (0.88 V), the SCoNC catalyst has a more positive half-wave potential (0.91 V) (Fig. 4f). The current density at 1.59 V approaches 50 mA cm^−2^, demonstrating higher OER activity in comparison with the benchmark IrO_2_ (E_j10_ = 1.62 V vs. RHE) (Fig. 4g). Additionally, it was indicated that the CoN_4_ sites on the carbon matrix were the most active sites for bifunctional oxygen electrocatalysis by constructing different CoN_x_ (x = 1, 2, 3, 4) models to investigate ORR and OER activities (Fig. 4h). The synergistic effect of the stable substrate and high concentration of active sites can be considered as an effective strategy to promote the bifunctional oxygen catalysis activity of M-N-C catalysts. Based on fixing the active material, the strategies (e.g., the introduction of heteroatoms in M-N-C materials, the introduction of defects, and the adjustment of multiple TMs) to modulate the atomic structure or chemical environment around the active site have been well proven in enhancing the bifunctional activity of M-N-C catalysts for ORR and OER.

### Heteroatom Coordination

Along with the advancement of atomically dispersed M-N-C catalysts, introducing non-metallic heteroatoms into carbon materials is one of the simplest methods to directly change the electron environment and atomic configuration to modulate the catalytic activity [18, 31, 103–107]. Besides N-doping, other heteroatoms (e.g., S, P, B, and O) can also provide sites to anchor isolated metal atoms, improving the metal atom deposition in carbon materials [54, 59]. In addition, they can destroy the electric neutrality of the carbon matrix and refine the charge and spin distribution to effectively form additional active sites, which will increase the amount and the reactivity of active sites simultaneously [5, 61, 75]. Based on pristine M-N-C catalyst, inserting secondary heteroatoms with a discrepancy in electron spin density and electronegativity can modulate the coordination environment and correspondingly alter electronic configurations of the M-N-C sites. Heteroatoms can also influence the catalytic activity of the M-N-C sites through the long-range delocalization and charge transfer effect, even if they cannot bind directly to the M-N-C sites [18, 61, 66, 76, 78, 97, 103, 104, 108]. Moreover, injecting heteroatoms into the electrocatalyst surface can significantly increase the density of active sites without severe structural collapse [12].

**Fig. 5:**
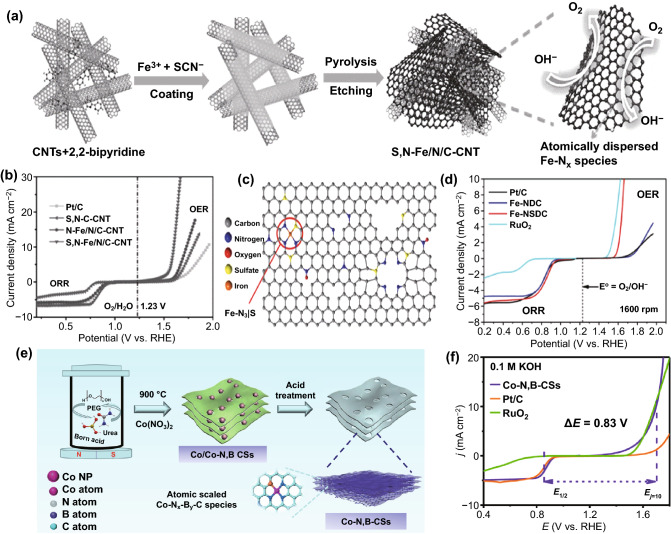
**a** The synthetic procedure for S, N-Fe/N/C-CNT. **b** LSV of all samples for ORR and OER in 0.1 M KOH [65]. Copyright 2017, Wiley–VCH. **c** Atomic structure model of Fe-NSDC. **d** Overall ORR and OER polarization curves of different catalysts in O_2_-saturated 0.1 M KOH [63]. Copyright 2019, Wiley–VCH. **e** Synthetic procedure of Co–N, B-CSs. **f** Overall polarization curves of different catalysts in 0.1 M KOH [64]. Copyright 2018, American Chemical Society

The M-N-C catalysts (especially for Fe-N-C) have been successfully used for ORR catalysis. However, their laggard OER activity results in a lethargic fashion for the charging of rechargeable ZABs [63]. Incorporating heteroatoms to enhance the bifunctionality of M-N-C has led to continuous exploration [63–65, 67, 106, 109–112]. It was reported that S was preferred in atomically dispersed Fe-N-C catalysts to promote the ORR activity, and heteroatoms (e.g., S and B) were active for OER in previous works [94, 97]. Chen et al. developed a single-atom bifunctional catalyst by preparing an atomically dispersed catalyst of Fe-N_x_ species on a layered carbon layer co-decorated with N and S (Fig. 5a) [65]. By introducing N and S, it is possible to lead to positively charged carbon atoms adsorbing oxygen by creating an uneven charge distribution. Meanwhile, thanks to the abundance of highly active atomically dispersed Fe-N_x_ sites, and the hierarchical structure that can expose more active sites, the obtained catalyst exhibits impressive bifunctional activity (ΔΕ = 0.75 V) for the rechargeable ZABs (Fig. 5b). In the study conducted by Zhang et al., the crucial role of S for the enhancement of ORR and OER activities was demonstrated further in the S-doped Fe-N-C electrocatalyst [63]. Atomically dispersed Fe is surrounded by three N atoms and one S atom by direct coordination, forming Fe-N_3_/S (Fig. 5c). S doping could optimize the charge distribution and spin distribution of Fe-N-C catalysts, which triggers the enhancement of OER activity. In addition, S doping enhances hydrophilicity and reduces charge transfer resistance. The resulting Fe-N_3_/S sites combine with Fe-N_x_ active sites to produce high-performance bifunctional catalytic activity (ΔΕ = 0.8 V) (Fig. 5d). Yang et al. discovered that the increased S atoms incorporation is an efficient way to increase the density of atomically distributed active sites and boost bifunctional catalytic efficiency [61]. Guo et al. proposed a B-doped Co-N-C bifunctional oxygen electrocatalyst by a soft template self-assembly pyrolysis approach (Fig. 5e) [64]. According to experiments and DFT calculations, it is confirmed that the B atom doping can improve electronic conductivity and generate more active sites by activating electron transfer near the Co-N-C site. It is noteworthy that the B-doped catalysts possess a lower onset potential than RuO_2_ and Pt/C and demonstrate excellent bifunctional electrocatalytic activity (ΔΕ = 0.83 V) (Fig. 5f). The synergistic effect of the active CoN_x_ species and the introduced phosphorus (P) atoms can also significantly increase the ORR and OER activities and durability [67].

**Fig. 6:**
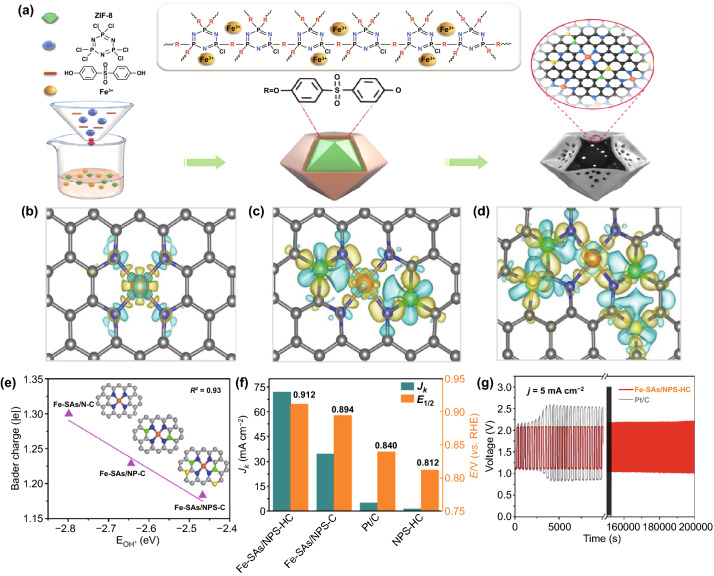
**a** Illustration of the preparation process of Fe-SAs/NPS-HC. Calculated charge density differences of **b** Fe-SAs/N-C, **c** Fe-SAs/NP-C, and **d** Fe-SAs/ NPS-C. **e** Linear relationship between OH* binding energy and Bader charge of single-atom iron in different samples. **f** Fe-SAs/NPS-HC and the corresponding reference catalysts were compared for J_k_ at 0.85 V and E_1/2_ in 0.1 M KOH. **g** Charge–discharge cycling performance of Zn-air batteries based on Fe-SAs/NPS-HC and Pt/C [112]. Copyright 2018, Nature Publishing Group Royal Society of Chemistry

Previous studies have provided convincing evidence that the synergistic effect of several heteroatoms can significantly increase the ORR/OER catalytic performance of carbon materials [106, 109–111]. However, catalytic performances are hindered primarily by unavoidable dispersion and uniformity of the different heteroatoms. Chen et al. developed a novel strategy to fabricate a Fe-SAs/NPS-HC catalyst with a functionalized hollow structure from a metal–organic framework@polymer composite (Fig. 6a) [112]. The functionalized hollow structure provides atomically distributed active sites and enhanced kinetics. The electronic modulation of active metal centers also strengthens catalytic activity, including close-range coordination with N and long-range interaction with S and P (Fig. 6b-d). As shown in Fig. 6e, Fe-SAs/NPS-C has the smallest OH* binding energy, showing the best “4e^−^ reduction” catalytic performance and kinetics. It is further demonstrated that Fe-SAs/NPS-HC achieves excellent ORR performance (Fig. 6f), due to structure functionalities and electronic control from surrounding S and P atoms. Zn-air batteries based on Fe-SAs/NPS-HC have good long-term durability with negligible voltage changes, even after 500 charge and discharge cycles tested within 200,000 s (Fig. 6g).

### Defect Engineering

**Fig. 7:**
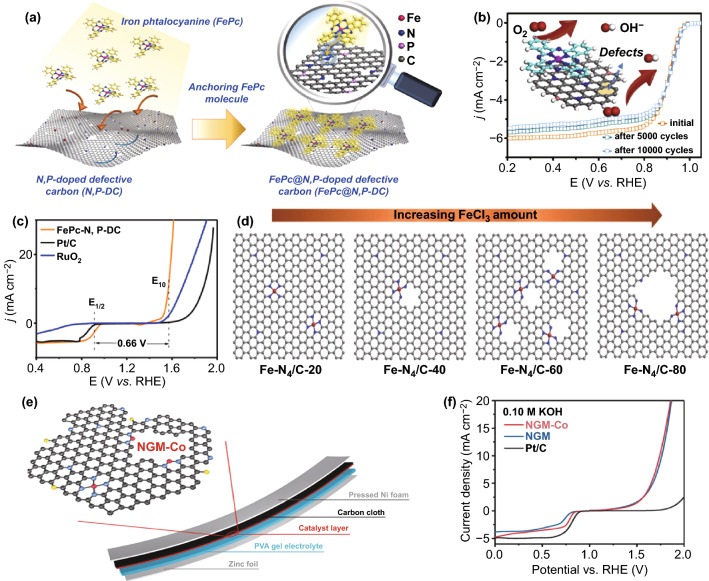
**a** Illustration of the preparation process of FePc@N, P-DC catalyst. **b** Overall polarization curves of different catalysts in 0.1 M KOH. **c** ORR LSV of FePc@N, P-DC measurement before and after 5000 and 10,000 cycles [68]. Copyright 2020, Elsevier. **d** Schematic diagram of morphology evolution of Fe-N_4_-C_-X_ samples with different amounts of reactant FeCl_3_ [113]. Copyright 2020, Wiley–VCH. **e** Schematic illustration of the flexible solid ZAB and the hierarchical NGM-Co catalyst. **f** Overall polarization curves of different catalysts in 0.1 M KOH [114]. Copyright 2017, Wiley–VCH

Intrinsic defects (such as topological defects, edge defects, and vacancy defects) in carbon catalysts have been gradually demonstrated to be an efficient, crucial strategy to contribute to enhancing the catalytic activities toward both ORR and OER [114–118]. The intentionally introduced defect sites to carbon materials can enhance the electrocatalytic activity under a similar mechanism of heteroatom doping. On the one hand, intrinsic carbon defects can directly serve as the potential active sites for ORR/OER by generating charge redistribution, altering the electronic structure, and modulating the adsorption free energy of critical intermediates [16, 78, 119, 120]. Specifically, both edge effects and topological defects are more favorable for both ORR and OER than heteroatom-doped sites [68, 121]. In addition, DFT calculations found that the carbon atoms located at the armchair edge and adjacent to the graphitic N dopants are the intrinsic active sites for ORR and OER [122]. On the other hand, defects on the surface of carbon (such as vacancy or topological defects) can act as “traps” to anchor single-atom metal, stabilize metal centers, and restrict undesirable agglomeration [5, 59, 75, 78, 120]. Taking advantage of the electronic redistribution and coordination environment of the stable M–N binding, atomically dispersed M-N-C centers on the defect “holes” become electrocatalytic active centers [68, 123]. By directly coordinating the metal-N_4_ organic macrocyclic molecules to defects in the carbon nanosheets, Cheng et al. developed an atomically dispersed M-N-C catalyst (Fig. 7a) [68]. The defective carbon boosts the high spin state of the Fe center, thus optimizing ORR performance (E_1/2_ = 0.90 V) and ensuring excellent cycling durability (Fig. 7b). In addition, it was demonstrated that topological defects coupled with the FeN_4_ sites could further enhance OER activity by comparing the OER activities of N, P-DC and FePc@N, P-DC. As shown in Fig. 7c, FePc@N, P-DC displays impressive excellent bifunctional electrocatalytic activities for ORR and OER (ΔE = 0.66 V). Wang et al. reported that the controllable e-ND-Fe configurations by constructing nitrogen-modified divacancies (ND) to trap atomic Fe species [113]. The gradual increase of FeCl_3_ can cause more hole defects; however, the further increase of Fecl_3_ leads to the collapse of the nanostructure (Fig. 7d). Tang et al. fabricated atomically dispersed Co-N_x_-C active sites on graphene by directly utilizing the intrinsic structural defects. Owing to the abundance of defective edges in graphene, cobalt atoms may readily couple with pyridine N, which will make Co-N_x_-C catalyst facilely achieve atomic dispersion and bifunctional catalytic performance (Fig. 7e, f) [114].

**Fig. 8:**
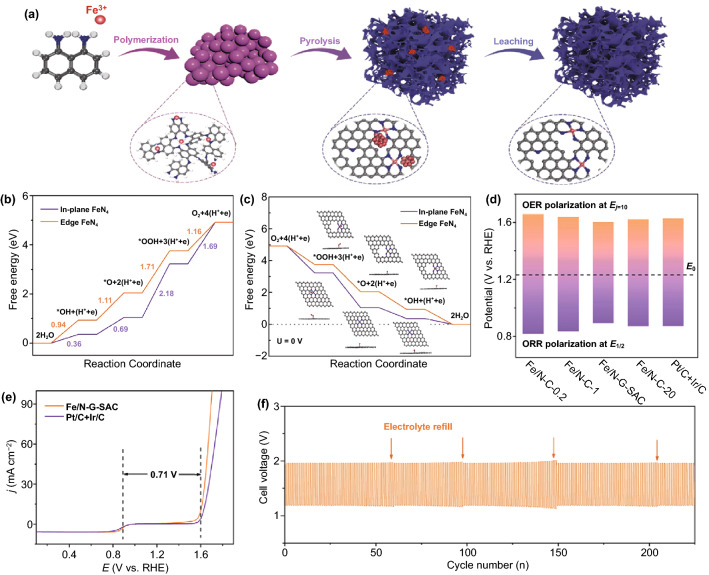
**a** The synthetic procedure of Fe/N-G-SAC. Gibbs free energy diagrams on the edge and in-plane sites for **b** ORR and **c** OER. **d** Potential gaps between the E_1/2_ and E_j10_ for different catalysts in 0.1 M KOH. **e** Overall LSV curves of Fe/N-G-SAC, Pt/C + Ir/C in 0.1 M KOH. **f** Charge and discharge cycling curves of the ZABs using Fe/N-G-SAC catalysts as the air cathode [23]. Copyright 2020, Wiley–VCH

Guided by the DFT prediction, atomically dispersed catalysts with enrich edge sites were prepared by self-sacrificed strategy. Mao et al. found that the combination of edge defects and FeN_4_ can boost the ORR activity of Fe–N/C electrocatalysts [124]. Xiao et al. developed a potentially scalable strategy to preferentially integrate FeN_4_ edge sites into the highly graphitic graphene sheet (Fig. 8a) [23]. DFT calculations confirmed that edge sites are superior to in-plane sites in ORR and OER catalysis. (Fig. 8b, c) The in situ generated Fe clusters lead to the formation of the desirable FeN_4_ edge sites and a highly ordered graphitic carbon structure, which suppressed electrochemical corrosion of carbon substrate and improved the distinguished stability under the harsh OER condition. A smaller value of ΔE as low as 0.71 V indicates better bifunctional catalytic performance than the commercial noble metal catalysts (0.756 V) and other counterparts (Fig. 8d, e). The long-term durability investigated by galvanostatic charge–discharge at a constant current density of 10 mA cm^−2^ reveals that Fe/N-G-SAC is a promising catalyst with excellent durability for the rechargeable ZAB application (Fig. 8f).

### Dual-Metal Sites

Recently, it has been demonstrated that the M-N-C with bimetal atoms can significantly improve the electrocatalytic performance compared with the single-metal M-N-C [53, 69, 125, 126]. The introduction of secondary metal atoms can raise the opportunity to increase flexibility and concentrate different activities toward ORR and OER [43, 127]. Furthermore, the synergistic effect between the two metallic elements can significantly increase their catalytic efficiency and stabilities by promoting electron transfer pathways and electron distributions [31, 43, 70, 95]. Considering the difference of the electronegativity for different metal atoms, the coordination of dual-metal-atom with N atoms makes it possible to optimize the electronic structure of the carbonaceous framework and the specific adsorption free energy for reactants [127]. Therefore, it inspires integrating metal atoms with different catalytic advantages into the same atom-dispersed N-doped carbon material to obtain a high-performance bifunctional electrocatalyst [70, 128]. In the past few years, atomically dispersed M-N-C materials with bimetal atoms have been studied for bifunctional oxygen catalyses, such as bimetallic Fe-Co [70, 128], Co-Ni [22], Co-Zn [95, 129], and Ni-Fe [21, 29, 130].

**Fig. 9:**
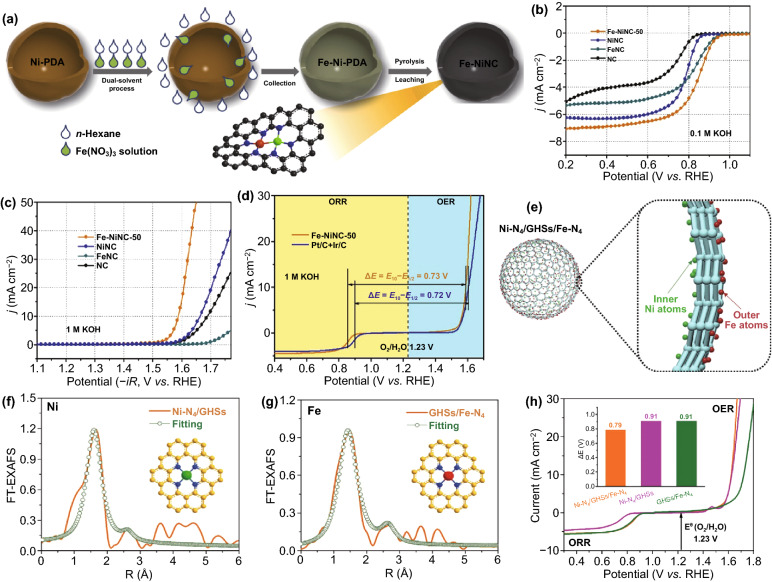
**a** The synthetic procedure of Fe-NiNC catalysts. LSV curves of the catalysts for **b** ORR in 0.1 M KOH, **c** OER in 1 M KOH, and **d** overall bifunctional performance in 1 M KOH [21]. Copyright 2020, Elsevier. **e** The structural diagram of the Ni-N_4_/GHSs/Fe-N_4_ catalyst. Atomic structure model of **f** Ni-N_4_/GHSs and **g** GHSs/Fe-N_4_. **h** Overall polarization curves of catalysts in 0.1 M KOH [29]. Copyright 2020, Wiley–VCH

FeNi catalysts with N-doped carbon anchored in an atomically dispersed form have been extensively studied for their high activity and stability [21, 29, 130]. Cheng et al. made Fe and Ni atoms in bimetallic FeNi catalysts stabilized via the coordination of N [130]. They found that bimetallic FeNi catalysts are superior to atomically dispersed single Fe and Ni catalysts in terms of reversible OER and ORR performance. Similar results were verified by Ma et al., Fe sites with neighboring Ni were responsible for both ORR and OER, as suggested by DFT simulations and in-situ Raman [53]. Recent studies have attributed the greater bifunctionality to synergistic interactions between bimetal atoms. However, there are great challenges yet to achieve the atomic control of targeted reactive sites comprising binary metal atoms and clarify the identification of the deeper functional mechanism of bimetallic atoms for boosting the reversible ORR/OER [22, 75, 95]. Zhu et al. successfully introduced atomically dispersed Fe-Ni sites onto the N-doped carbon hollow sphere (Fig. 9a) [21]. By redistributing the charge between the Fe and Ni, Fe-Ni binding may exhibit mutual enhancement upon Fe and Ni atoms, making the Fe atoms and Ni atoms as the active sites for ORR and OER, respectively. As expected, Fe-NiNC-50 exhibits better bifunctional oxygen activity than NiNC, FeNC, and the mixture of NiNC and FeNC, providing additional evidence that Fe-Ni bonds play a crucial role in oxygen electrocatalysis (Fig. 9b-d). Yu et al. reported that the Ni site regulates the electronic structure of the Fe site based on the density functional theory calculations, which further reduces the energy barrier of the rate-limiting step. Furthermore, it was confirmed that the Fe–Ni-N_6_ structure in the dual-sites single-atom catalyst has high intrinsic reactivity and structural stability [131].

In the atom-dispersed catalyst, bimetals can not only be connected through chemical bonds but also exist in coordination with four N atoms via the formation of two types of single atoms (Ni-N_4_ and Fe-N_4_), respectively. Chen et al. developed a new single-atom functional Janus hollow graphene to allocate atomically dispersed Ni and Fe on the inner and outer wall (Fig. 9e-g) [29]. It was demonstrated that the outer Fe-N_4_ clusters are responsible for excellent activity toward the ORR; however, the inner Ni-N_4_ clusters dominantly contribute to high activity toward the OER, which results in excellent electrocatalytic selectivity and bifunctionality. Compared with Ni-N_4_/GHSs (0.91 V) and GHSs/Fe-N_4_ (0.91 V) catalysts, the Ni-N_4_/GHSs/Fe-N_4_ electrode has superior bifunctional activity with an ∆E value of approximately 0.79 V (Fig. 9h). With Ni-N_4_ and Fe-N_4_ species embedded on different sides of hollow graphene to reduce active site aggregation, the high utilization efficiency of sites can be achieved. The separation of ORR and OER active sites can balance the competition between the two kinds of oxygen catalysis reaction rate-limiting steps by reducing the mutual interference.

### Porous Structure Design

There are general two ways to improve the bifunctional ORR/OER activity, which are enhancing their intrinsic activity and increasing the number of active sites exposed by the electrode [11]. Considering that both OER and ORR reactions occur on the catalyst surface, it is, therefore, essential to consider both the mass transport and electron transfer transmission [31]. Designing favorable hierarchical pore structures is also significant to boost bifunctional catalytic activity as it facilitates the utilization efficiency of active sites and accelerates mass transportation [112]. The hierarchically porous structure tends to significantly increase the surface area, and then amplify the density of active sites and expose each active site accessible to the reactants [12]. In addition to the specific surface area, a well-developed network with interconnected fractal pore structure and continuous multiscale channels is also critical for decreasing the diffusion resistance and facilitating the mass transfer and electron transport, ultimately ensuring the continuous proceedings of the catalytic reaction [43, 82, 128, 134].

**Fig. 10:**
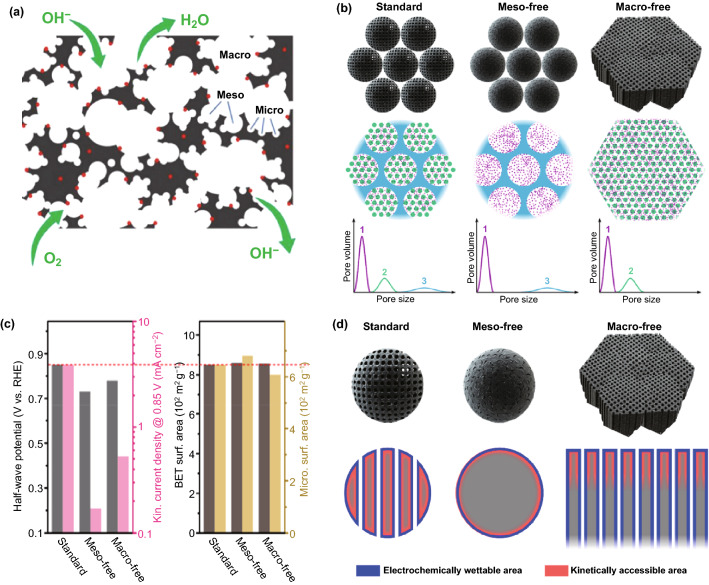
**a** Schematic diagram of hierarchically porous structures [132]. Copyright 2019, Royal Society of Chemistry. **b** Three N-doped carbon model catalysts “standard”, “meso-free” and “macro-free”. **c** ORR activity, BET and micropore surface areas of three N-doped carbon model catalysts. **d** Schematic illustration of electrochemically wettable and kinetically accessible area for three model catalysts [133]. Copyright 2019, American Chemical Society

Hierarchical pore structures with micropores, mesopores, and macropores of the electrocatalysts are considered as highly trusted nanostructures for exposed number/ density of the active sites and fast mass/electron transport (Fig. 10a) [12, 56, 132]. Micropores constitute a significant part of the specific surface area of catalysts, which can hold plentiful ORR/OER active sites [73, 134]. Oxygen molecules can be captured by micropores, making them more effectively combined with catalytically active sites [12]. Unfortunately, the presence of micropores cannot solely meet the rapid mass transfer needs of ORR and OER, which will result in limited electrocatalytic performance [132]. The existence of abundant large mesopores can facilitate electrolyte wetting of the solid surface area, promote accessibility of the dispersed active sites, and accelerate mass transport by reducing and smoothing the diffusion pathways [82, 132, 135]. Compared to the micro/mesoporous disordered channels, the interconnected ordered macropores can offer greater mass transport effectiveness and allow the exposure of a large number of active sites [136]. Specifically, macropores provide large-sized space for the transport of oxygen catalysis-related reactants to active sites, which facilitates the transfer of reactants and products and maintains the high-flux mass transport of the electrocatalytic process, especially under high current density [82]. The introduction of macropores to build hierarchically porous structures is meaningful for commonly employed MOF-derived electrocatalysts [132]. Lee et al. systematically compared the effect of its porous structure on its catalytic activity by developing three N-doped carbon model catalysts (“standard”, “meso-free” and “macro-free”) (Fig. 10b) [133]. The study found that ORR activities have little correlation with BET and micropores surface area but are related to the conditions for the full utilization of the active sites, including the electrolyte wetting and mass transport kinetics (Fig. 10c, d). Macro- and mesoporous structures contribute to different stages of reaction kinetics in ORR catalysis, making the systematic control of pore size distribution crucial to catalyst activity.

**Fig. 11:**
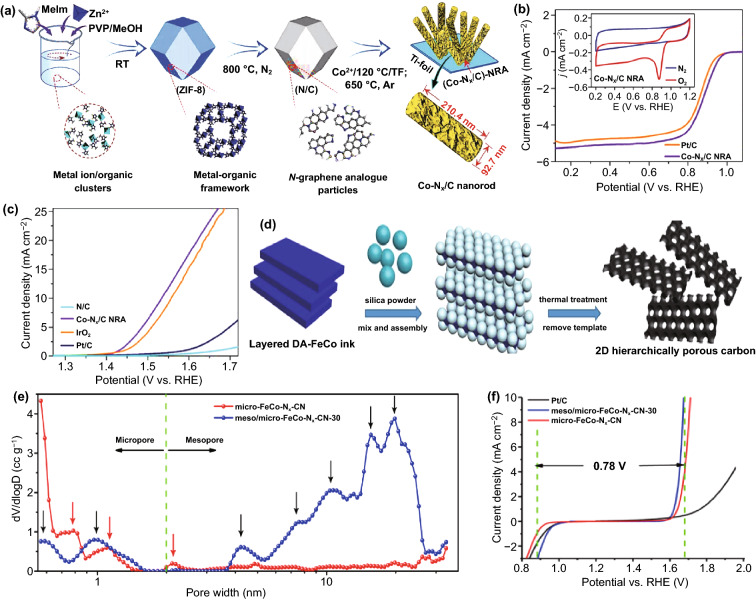
**a** Synthetic procedure for the Co-functionalized carbon nanorod array. **b** LSV curves of Co-N_x_/C NRA and contrastive catalysts for ORR in 0.1 M O_2_-saturated KOH. **c** LSV curves of Co-N_x_/C NRA and IrO_2_ for OER before and after 10,000 potential sweeps [71]. Copyright 2018, Wiley–VCH. **d** The synthetic procedure of and **e** pore size distribution curves of meso/micro-FeCo-N_x_-CN-30. **f** Overall polarization curves of different catalysts (ORR in 0.1 M KOH, OER in 0.1 M KOH) [70]. Copyright 2018, Wiley–VCH

Hence, it is essential to regulate both the pore size and structure (e.g., pore continuity, length, and pore size distribution) of bifunctional catalysts to get more exposed active sites, faster electron/mass transportation pathways, and higher surface areas [137]. However, disordered structure and a large number of blocked pores attacked by caustic and oxidizing conditions will collapse the electrode structure and seriously impede the transport efficiency, resulting in a loss of a large number of gas transport channels and active sites [12]. It is, therefore, necessary for an interface to be designed rationally with good channel connectivity and organized structure. According to many reports, the structure of interconnected fractals and ordered arrays have been considered the most promising architectures. The ordered array configuration allows lower transfer resistance, while the interconnected fractal structure ensures homogeneous transfer and conduction properties [128]. For creating pores with consistent size and architecture, template methods are most popular and straightforward [82]. Using organometallic synthesis, Mu et al. fabricated an advanced Co-N_x_/C nanorod array (Fig. 11a). Remarkably, as a bifunctional catalyst, the impressive catalytic performance (ΔE ≈ 0.65 V) is primarily attributed to the synergistic effect of the abundant Co–N active sites and the unique nanorod geometry with enriched porosity and high surface area (Fig. 11b, c) [71]. Li et al. gave an in-depth analysis of the influence of nanoarchitecture and microporosity in carbon materials on reversible oxygen catalytic activity and stability [70]. A facile method for synthesis of meso/micro-FeCo-N_x_-CN as a high-performance bifunctional oxygen catalyst is using a salt template and silica template simultaneously (Fig. 11d). The results of analyzing the pore size distribution by the QSDFT model show that the addition of the silicon template increases the ratio of mesopores to micropores, even though the surface area is not significantly affected (Fig. 11e). The hierarchical structure and enriched reversible oxygen electrocatalytic sites attribute to the remarkable bifunctional activity (ΔE = 0.78 V) of meso/micro-FeCo-N_x_-CN (Fig. 11f). Furthermore, a charge–discharge test to determine the stability of the ZAB equipped with meso/micro-FeCo-N_x_CN lasts for over 40 h and the voltage remains fairly constant.

## Summary and Perspective

The past twenty years have witnessed the great progress of electrocatalysts for ZABs. The emergence of atomically dispersed catalysts with high atom utilization efficiency and tunable active sites has reinvigorated research targeting the discovery of low-cost electrocatalysts for oxygen catalysis. This review surveys the general principles for designing atomically dispersed M-N-C. The endeavors to enhance the intrinsic activity for ORR and OER are summarized including designing appropriate metal centers, introducing heteroatoms, manufacturing defects, and structuring bimetals active sites. Moreover, architecting favorable hierarchical-pores structures with a high specific surface area can facilitate the utilization efficiency of active sites and accelerate mass/electron transportation. Despite the achievements so far, there are still many challenges remaining to be addressed to develop highly efficient ZAB systems in the future. Thus, those challenges and perspectives are discussed as follows:

1. An in-depth understanding of the bifunctional reaction process is required. The complicated mechanisms of OER and ORR are strongly determined by different catalytic environments, raising intricate debates on the understanding of the real active sites for the attribution of bifunctional catalysts. Considering that single metal atoms, defects, and heteroatoms can act as active sites independently or cooperatively, the superior catalytic performance of atomically dispersed M-N-C is always ambiguously defined as a “synergistic effect”. However, the potential “synergistic effect” or even “inhibited effect” between different active sites shall be carefully considered. Previous studies have shown that the coordination structure and electronic configuration of metal atoms change dynamically during the reaction, and even morphology/composition reconstruction. It is necessary to measure and track the catalyst surface reconstruction process in time to capture the dynamic evolution of the bifunctional catalyst, which provides new insights for accurately identifying the real active sites on the electrocatalyst surface and revealing the catalytic mechanism. Given this, greater efforts should go toward combining in-situ/operando characterization technologies (such as X-ray absorption near-edge spectroscopy (XANES), extended X-ray absorption fine structure spectroscopy (EXAFS), and surface-enhanced Raman spectroscopy (SERS)) and theoretical calculations to accurately capture the dynamic reconstruction of the electrocatalyst, identify active sites and understand the reaction mechanism. It will provide the guidelines to finely tune the properties and activities of M-N-C catalysts with optimal activity, selectivity, and stability, which is crucial for practical applications.
2. It is still challenging to fabricate single metal atoms dispersed on the porous N-doped carbon with a precise coordination structure and high metal loading. Despite the widespread availability of the pyrolysis process for the fabrication of the atomically dispersed M-N-C, the precise synthesis at the atomic level is a major challenge. This is because pyrolysis usually collapses the pre-designed precursor structure and forms the undefined structure of the M-N-C active sites. Based on this fact, it is critical to promote reliable strategies and synthetic methods that can precisely tune the atomically dispersed active sites. MOFs are considered the most promising precursors to developing atomically dispersed M-N-Cs. However, MOFs are extremely expensive, complex to synthesize, and low yield, significantly limiting their application on a large scale. It is desirable to enhance the catalyst activities by combining MOFs with other low-cost precursors to enrich the single atomic site population within M-N-C catalysts, by increasing their metal loadings. However, obtaining high metal loading without aggregation for increasing the density of the active site is another challenge. It may be effective to solve this problem by rationally designing hierarchical porous structures with suitable pore-size distribution to increase metal doping content, which can also provide good mass transport/electron transfer. The synthesis of hierarchical porous structures usually requires complex procedures with high cost. Therefore, it is necessary and urgent to develop a low-cost and simple synthesis method to produce high-metal-loaded atom-dispersed catalysts on a large scale.
3. The challenge of the insufficient stability of the bifunctional M-N-C catalysts for long-term operation remains, particularly in cases of the OER process. After repeated deep charging and discharging of the rechargeable ZABs, the conductivity and hydrophobicity of the air electrode are severely reduced. In this case, surface/interface nanoengineering should be used to maintain the stability of rechargeable ZABs. For the carbon material with a low graphitization degree during the charging process, a large number of defects are susceptible to corrosion at high overpotentials, which causes a serious performance decay of rechargeable ZABs. To address this problem, raising the graphitization degree of a stable carbon structure through heat treatment and stable heteroatoms doping can increase the stability of M-N-C catalysts.
4. In addition to improving the activity and stability of bifunctional catalysts, upgrading the battery configuration of ZABs can also not be ignored. Depending on the state of the air electrode in ZABs, the mechanism of ORR and OER could be influenced by the different conditions, including the irreversible deposition and accumulation of ZnO, the formation of Zn dendrites, the high concentration of electrolyte, and the configuration of batteries. The deposition of ZnO can block the pores of the air electrode and cover the active sites, which will prevent the diffusion of reactive intermediates and finally cause premature battery death. Battery performance is also significantly affected by alkaline electrolyte loss and degradation. To guarantee the stable and durable charge–discharge operation of ZABs for practical application, there are still many important topics including composition modification of the Zn electrodes, constructing a protective layer to inhibit the excessive enrichment of ZnO on the air electrode, and adopting a flowing-electrolyte design. For all-solid-state ZABs, the backward development of high-performance electrolyte membranes has also severely restricted their applications. In addition to the formation of dendrites and surface passivation at the electrolysis-zinc electrode interface, the resistance at the air electrode–electrolyte interface is much higher than that of the water interface, which severely limits the performance of solid-state ZABs. Exploring solid electrolytes with high ionic conductivity and mechanical strength, better water absorption and retention, and good safety and stability and solving the problems of the electrolyte–electrode interface are necessary to meet the commercial needs of solid-state ZABs. For flexible ZABs, flexible collectors, flexible electrolytes, and appropriate packaging technologies should all be considered and fully optimized to maintain stable electrochemical performance for a long time under bending conditions.
